# SARS-CoV-2 antibodies in employees working in non-medical contact-intensive professions in the Netherlands: Baseline data from the prospective COco-study

**DOI:** 10.1016/j.pmedr.2021.101594

**Published:** 2021-10-08

**Authors:** Dymphie Mioch, Sandra Kuiper, Wouter van den Bijllaardt, Cornelia H.M. van Jaarsveld, Jan Kluytmans, Esther Lodder, Michel D. Wissing, for the COco study group

**Affiliations:** a Regional Public Health Service (GGD) of West-Brabant, Breda, The Netherlands.; b Department of Infection Control, Amphia Hospital, Breda, the Netherlands; Microvida Laboratory for Medical Microbiology, Amphia Hospital, Breda, The Netherlands; c Radboud University Medical Center, Radboud Institute for Health Sciences, Department of Primary and Community Care, Nijmegen, The Netherlands; d Julius Center of Health Sciences and Primary Care, UMC Utrecht, University Utrecht, Utrecht, The Netherlands

**Keywords:** COVID-19, SARS-CoV-2, Coronavirus, COVID-19 serological testing, Seroepidemiologic studies, Non-medical contact-intensive profession, Hospitality industry, Hairdressers

## Abstract

COVID-19 has made a global impact since early 2020, requiring characterization of the SARS-CoV-2 virus, including transmission risk. The COco-study aims to evaluate the risk for COVID-19 infections in two non-medical contact-intensive professions. COco is a prospective cohort study evaluating SARS-CoV-2 antibodies in hairdressers and hospitality personnel in the province of North-Brabant in the Netherlands, using a total antibody enzyme-linked immunosorbent assay. Baseline data from June/July 2020 were analyzed. Participants filled out a questionnaire, providing information on demographics, health, work situation, and risk factors for COVID-19. Odds ratios (OR) and 95% confidence intervals (95% CI) were calculated using logistic regression. In June/July 2020, 497 participants were enrolled: 236 hairdressers, 259 hospitality employees, and two participants worked in both industries. Hospitality staff was more frequently seropositive than hairdressers (14.2% versus 8.0%, respectively; OR 1.9, 95% CI 1.1–3.4). Furthermore, a high education level (OR 3.0, 95% CI: 1.7–5.6) and increased alcohol use (OR, 7 glasses per week increment: 1.3, 95% CI: 1.1–1.5) were associated with seropositivity. Of the 56 seropositive participants, 18 (32%) had not experienced any COVID-19 symptoms. The symptoms anosmia/ageusia differed most evidently between seropositive and seronegative participants (53.6% versus 5.7%, respectively; P < 0.001 (chi-squared test)). In conclusion, four months after the first identified COVID-19 patient in the Netherlands, employees in the hospitality industry had significantly more frequently detectable SARS-CoV-2 antibodies than hairdressers.

## Highlights

• COco is a prospective cohort study conducted in the Netherlands, 2020–2021.
• COco evaluates COVID-19 transmission risk in non-medical contact professions.
• Hospitality staff had more frequently SARS-CoV-2 antibodies than hairdressers.
• Anosmia and ageusia were the most important symptoms associated with seropositivity.
• A high education level and alcohol use were also associated with seropositivity.

## 1 Introduction

On February 27th, 2020, the first Dutch citizen was diagnosed with COVID-19, caused by Severe Acute Respiratory Syndrome Coronavirus 2 (SARS-CoV-2) (Gorbalenya et al., 2020). The first wave hit the Netherlands in March 2020. To estimate the percentage of a population that has been infected with the SARS-CoV-2 virus, serology studies are conducted to measure antibodies against SARS-CoV-2 (Anand et al., 2020, Vos et al., 2020). In April 2020, a seroprevalence of 2.7–2.8% was reported in the Dutch population (Vos et al., 2020, Slot et al., 2020), which increased to 4.5–5.5% in May-July (Sanquin Research, 2020, PIENTER Corona study, 2020).

A partial lockdown was implemented in the Netherlands in mid-March, including measures such as physical distancing and closure of some businesses (Government of the Netherlands, 2020). Subsequently, transmission decreased in the Netherlands, as in most other high-income countries. However, little is known about the efficacy of individual components of the strategies used, and/or the contribution of seasonal changes (Jones et al., 2020, Smit et al., 2020, Bendavid et al., 2021). One component was the closure of non-medical contact-intensive professions, such as the hospitality industry and hairdressers. While some studies have suggested that these businesses may have contributed significantly to COVID-19 outbreaks, the extent of their contribution remains unknown, while closure of such businesses had large impacts on society. Furthermore, it is unknown whether employees in such professions are at increased risk for COVID-19. Considering that employees risk being in close contact with customers or colleagues infected with SARS-CoV-2, one would expect that they have an increased risk, and subsequently form a risk for transmission to their colleagues and customers, particularly when being a-/ presymptomatic but contagious.

For abovementioned reasons, a prospective cohort study evaluating antibody levels against SARS-CoV-2 in employees working in two non-medical, contact-intensive professions, namely hairdressers and the hospitality industry (e.g. bars, restaurants, casinos), was initiated in June 2020 (the COco-study). We evaluate the percentage of employees infected with SARS-CoV-2 by measuring antibodies, while collecting various data on transmission risk via questionnaires.

## 2 Methods

### 2.1 Study design and population

The COco-study is a prospective cohort study. Its primary objective is to evaluate whether hairdressers and/or hospitality personnel have a significantly higher chance for SARS-CoV-2. For this purpose, baseline seroprevalence was measured in June-July 2020.

The study is being conducted in the western part of the province of North-Brabant in the Netherlands. This province had the highest COVID-19 incidence during the first wave in the Netherlands (PIENTER Corona study, 2020, National Institute for Public Health and the Environment (RIVM), 2021). Participants were eligible when working as hospitality staff or hairdressers in this region (Breda, Roosendaal and surrounding municipalities) for ≥ 100 h during the 3 months before enrolment. People were excluded if their age was < 18 years, if they were reluctant to venepuncture, incapacitated or unwilling to give informed consent, or a blood or plasma donor. The latter exclusion criteria was included since we are planning to compare seroprevalence in our cohort to seroprevalence in a matched cohort of blood and plasma donors in the region (Sanquin Research, 2020).

Recruitment started on June 1, 2020, and was completed on July 14. Hospitality personnel was primarily recruited via the national organization representing hospitality businesses (Koninklijke Horeca Nederland, KHN). This organization contacted all hospitality businesses in the region to inform them about our study protocol. Participants, both hospitality staff and hairdressers, were also recruited via social media, the website of the regional public health service (Gemeentelijke Gezondheidsdienst, GGD) of West-Brabant, and word by mouth advertising. Additionally, hairdressers were recruited by distribution of flyers. For hairdressers, we ensured inclusion of hairdressers in large companies, in ‘mon-and-pop’ stores, and hairdressers who do not own a store but cut hair from their home or their customer’s home. As more people volunteered than our required sample size, we applied ‘first come, first serve’.

We aimed to recruit 238 hairdressers and 260 hospitality personnel, based on our power calculation (supplementary data). The number of participants working in the hospitality industry was higher, because we expected a higher dropout rate in this industry due to job changes.

The COco-study has been approved by the Medical research Ethics Committees United (MEC-U) at Nieuwegein (project number A20.247/R20.041). It follows laws and guidelines on research with human subjects, including international standards such as the Declaration of Helsinki. Participation was voluntary after providing written informed consent. Data were stored pseudonymously using a study code; individual participants could not be identified by this code and only researchers involved with the COco-study had access to these data.

### 2.2 Data collection and analyses

During venepuncture, 3.5 ml blood is drawn and analyzed by the Microvida Laboratory for Medical Microbiology, location Amphia Hospital. The Wantai SARS-CoV-2 antibody (Ab) ELISA (Wantai Biological Pharmacy Enterprise Co., Ltd., Beijing, China) was performed following manufacturer’s instructions on a DS2, an open ELISA processing platform (Dynex technologies, Chantilly, VA, USA). The Wantai SARS-CoV-2 antibody (Ab) ELISA is a qualitative double-antigen sandwich immunoassay that detects all SARS-CoV-2 immunoglobin isotypes (IgA, IgM, or IgG) against the receptor binding domain of the spike protein. It has an estimated sensitivity of 96% and specificity of 99% (Self et al., 2020). Samples were considered positive when the signal to cut-off ratio was > 1.0. The highest signal to cut-off ratio detectable was 13.2: stronger signals were registered as 13.2.

Questionnaires were sent at baseline (see supplementary data). We analyzed data from the baseline questionnaire, using the variables: work setting (hairdresser, hospitality industry), job position (various categories), age (in years), sex (male/female), born in the Netherlands (yes/no), household size (1–15 persons), education level (low, middle, high), financial difficulties (yes/no), workplace location (by municipality), working hours (per week), chronic disease (yes/no), body-mass index (BMI), smoking status in 2020 (yes/no), smoking quantity (number of cigarettes per day), alcohol use in 2020 (yes/no), alcohol quantity (number of glasses per week), and reported symptoms related to COVID-19 (yes/no, per symptom). A participant was considered to have a chronic disease if he/she mentioned a chronic disease or listed medication which is used for chronic diseases only. BMI was calculated by weight in kilograms divided by height in meters squared; a participant had a normal weight, overweight, or obesity if the BMI was < 24.9 kg/m^2^, 25.0–29.9 kg/m^2^, >30.0 kg/m^2^, respectively. Education level was divided into three categories (low, middle, high): participants were considered to have received low education when the level of completed education was primary or pre-vocational secondary education, middle education when participants completed vocational secondary education or post-secondary education not at a university level (such as a hairdressing school), and high education when the participant had a college degree or higher. Some participants did not provide a single number to describe their average number of working hours per week. If they provided a range, its mean was registered; if they provided a minimum number of hours, this minimum was registered. One participant provided ‘normal week’ as an answer, which we labelled as unknown.

### 2.3 Statistical analyses

Descriptive statistics and frequencies were used to analyze baseline characteristics. Baseline characteristics were compared using chi-square tests for dichotomous categorical variables and Wilcoxon rank-sum tests for numerical variables, due to the non-normal distribution of numerical variables. The ordinal categorical variable education (low, middle and high) was analyzed using a Wilcoxon rank-sum test. Chi-squared tests were used to compare COVID-19 related symptoms between seropositive and seronegative participants. Uni- and bivariable logistic regression models were used to calculate odds ratios and their respective 95% confidence intervals for variables associated with seroprevalence of SARS-CoV-2 antibodies. All assumptions for binary logistic regression were met. Due to limited statistical power, multivariable analyses were limited to one covariate: when conducting multivariable analyses with multiple covariates, odds ratios remained similar, but confidence intervals widened significantly (data not shown). Hence, all covariates were analyzed in bivariable analyses. Numerical variables were analyzed as continuous variables to attain highest statistical power; categorical variables were entered as dichotomous variables. Participants with missing data were excluded from analyses; this applied to only one participant who had an unknown number of working hours. P-values below 0.05 were considered statistically significant in all analyses. All analyses were conducted using SPSS Statistics 24.0.

## 3 Results

**Fig. 1:**
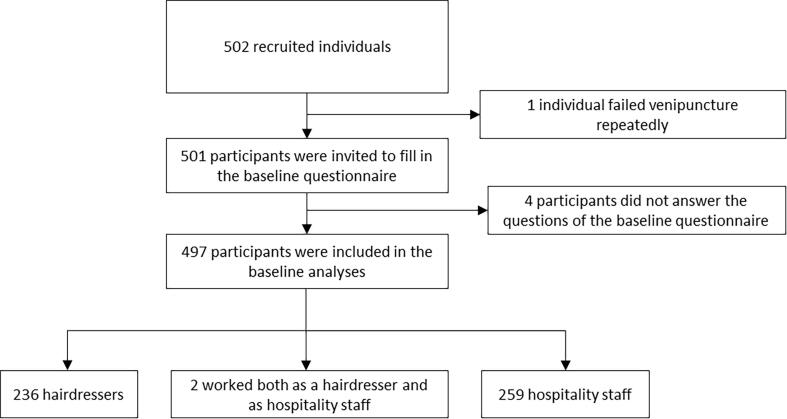
Flow chart.

In total, 502 individuals were recruited for the COco-study (Fig. 1). Five participants were excluded resulting in a baseline cohort consisting of 497 participants, of whom 259 individuals who worked in the hospitality industry, 236 as hairdressers and 2 individuals who worked both in the hospitality industry and as a hairdresser (Fig. 1). Most hospitality staff was working as a manager (41.8%) or in service (41.0%). For the hairdressers, 99.2% of participants cut hair of customers, either as an owner or an employee. Baseline characteristics are summarized in Table 1. The majority of participants were women (72.4%), particularly amongst hairdressers (89.9%). Median age was 38 years (range 17–74 years) in the full cohort. Hairdressers were generally older compared to hospitality personnel (median age 41 versus 32 years, P < 0.001). Most hairdressers (80.3%) had a middle-level education; in the hospitality industry 50.2% had a middle-level education while 31.0% had a higher-level education. Due to different recruitment strategies, 88.1% of hospitality employees worked in the cities of Breda and Roosendaal, while 53.0% of the hairdressers worked in these cities. Most participants were born in the Netherlands (94.8%) and had no chronic disease (71.2%). Hospitality staff smoked more often (31.8% versus 21.8%, P = 0.012), but if hospitality staff or hairdressers smoked, they smoked a similar number of cigarettes per day (11.5 versus 10.0 cigarettes per day, respectively; P = 0.79). Alcohol use was higher amongst hospitality staff (87.0%) compared to hairdressers (79.8%; P = 0.031). Furthermore, hospitality staff that used alcohol, drank on average 10 alcohol units per week, versus 3 units for hairdressers (P < 0.001).

**Table 1:** Baseline characteristics of participants in the COco-study.

		Total group (N = 497)		Hospitality industry (N = 261)		Hairdresser industry (N = 238)		P-value
		n	%	n	%	n	%	
Sex	Men	137	27.6	114	43.7	24	10.1	**<0.001**
	Women	360	72.4	147	56.3	214	89.9	
Age (in years)	Median (Min–Max)	38.0 (17–74)		32.0 (17–67)		41.0 (18–74)		**<0.001**
	17–29 years	169	34.0	118	45.2	52	21.8	
	30–50 years	214	43.1	82	31.4	132	55.5	
	51–74 years	114	2.9	61	23.4	54	22.7	
Born in								
the Netherlands	Yes	471	94.8	246	94.3	227	95.4	0.573
Household size	Median (Min–Max)	3.0 (1–15)		3.0 (1–15)		3.0 (1–6)		**0.001**
	1-person (participant lives alone)	50	10.1	34	13.0	16	6.7	
	2-persons	141	28.4	91	34.9	50	21.0	
	3-persons	120	24.1	51	19.5	70	29.4	
	4-persons	134	27.0	55	21.1	79	33.2	
	5-persons	38	7.6	18	6.9	21	8.8	
	>5 persons	14	2.8	12	4.6	2	0.8	
Education level	Low	88	17.7	49	18.8	40	16.8	**<0.001**
	Middle	321	64.6	131	50.2	191	80.3	
	High	88	17.7	81	31.0	7	2.9	
Financial difficulties	Yes	88	17.7	59	22.6	29	12.2	**0.002**
Workplace location	Breda	272	54.7	176	67.4	97	40.8	**<0.001**^b^
	Roosendaal	83	16.7	54	20.7	29	12.2	
	Oosterhout	26	5.2	5	1.9	21	8.8	
	Etten-Leur	25	5.0	5	1.9	20	8.4	
	Other municipalities	91	18.3	21	8.0	71	29.9	
Working hours	Median (Min–Max)	32.0 (8–100)		32.0 (8–100)		30.0 (8–65)		0.102
	8–20 h	119	24.0	76	29.1	44	18.5	
	21–40 h	256	51.6	103	39.5	154	64.7	
	≥ 40 h	121	24.4	81	31.0	40	16.8	
	Unknown	1	0.2	1	0.4	0	0.0	
Chronic disease^a^	Yes	143	28.8	70	26.8	74	31.1	0.292
BMI	Median (Min–Max)	24.4 (16.0–43.8)		24.1 (16.1–41.1)		24.5 (16.0–43.8)		0.158
	Normal weight	288	57.9	154	59.0	136	57.1	
	Overweight	145	29.2	79	30.3	66	27.7	
	Obesity	64	12.9	28	10.7	36	15.1	
Current smoker	Yes	134	27.0	83	31.8	52	21.8	**0.012**
Current smokers: number of cigarettes per day	Median (Min–Max)	10.5 (1–30)		11.5 (1–30)		10.0 (1–25)		0.785
	< 10	48	35.8	33	39.8	16	30.8	
	10–20	78	58.2	43	51.8	35	67.3	
	> 20	8	6.0	7	8.4	1	1.9	
Current alcohol use	Yes	415	83.5	227	87.0	190	79.8	**0.031**
Current alcohol users: alcohol units per week	Median (Min–Max)	6.0 (0.5–73)		10.0 (0.5–73)		3.0 (0.5–49)		**<0.001**
	0.5–7	222	53.6	86	37.9	137	57.6	
	8–14	102	24.6	64	28.2	39	16.4	
	15–21	46	11.1	38	16.7	8	4.2	
	> 21	45	10.8	39	17.2	6	3.2	

Baseline characteristics of the two study groups, hospitality personnel and the hairdressers, were compared using Wilcoxon Rank Sum tests (numerical variables) or chi-squared tests (categorical variables). P-values below 0.05 were considered statistically significant and marked bold.

All values are n (%) unless specified otherwise.

a Patients were considered to have a chronic disease if they reported a chronic illness and/or were chronic medication users.

b For workplace location, we compared those working in Breda/Roosendaal to those working in other cities or villages.

In total, 11.3% (56/497) of participants tested positive for SARS-CoV-2 antibodies. We first studied which symptoms, experienced in 2020 until the baseline measurement, were associated with seropositivity (Table 2). 202 patients mentioned symptoms that could have been related to COVID-19. Of those, 19% (n = 38) were seropositive. Eighteen of the 56 seropositive participants (32.1%) did not report a symptom, suggesting asymptomatic infections or recall bias.

**Table 2:** A comparison of symptoms in seropositive and seronegative participants of the COco-study.

Reported symptoms	Seropositive (N = 56)		Seronegative (N = 441)		P-value
	n	%	n	%	
Symptoms related to COVID-19	38	67.9	164	37.2	**<0.001**
Headache	36	64.3	203	46.0	**0.010**
Fatigue	32	57.1	245	55.6	0.822
Anosmia/ageusia	30	53.6	25	5.7	**<0.001**
Runny nose / nasal congestion	30	53.6	241	54.6	0.879
Coughing	27	48.2	205	46.5	0.807
Feeling feverish	22	39.3	74	16.8	**<0.001**
Recorded fever	21	37.5	99	22.4	**0.013**
Shortness of breath	20	35.7	107	24.3	0.064
Gastrointestinal symptoms	18	32.1	122	27.7	0.483
Loss of appetite	15	26.8	58	13.2	**0.007**
Severe, unexpected muscle or joint pain	14	25.0	67	15.2	0.061
Sore throat	14	25.0	154	34.9	0.139
General malaise	9	16.1	23	5.2	**0.002**
Pain during breathing	8	14.3	29	6.6	**0.038**
Confusion/irritability	5	8.9	28	6.3	0.465

Participants reported symptoms they had experienced from January 2020 until the time of baseline serology measurement. Percentages were calculated by dividing the number of seropositive/-negative persons with symptoms by the total number of seropositive/-negative persons, respectively (n/N). Symptoms of seropositive participants were compared to seronegative participants. P-values were calculated using a chi-squared test; P-values below 0.05 were considered statistically significant and marked bold.

When evaluating specific symptoms, anosmia/ageusia (loss of smell/taste) differed most evidently between seropositive and seronegative participants (53.6% versus 5.7%, respectively; P < 0.001). Furthermore, seropositive individuals reported more frequently recorded fever (37.5% versus 22.4%, P = 0.013), feeling feverish (39.3% versus 16.8%, P < 0.001), pain during breathing (14.3% versus 6.6%, P = 0.038), general malaise (16.1% versus 5.2%, P = 0.002), headaches (64.3% versus 46.0%, P = 0.010), and loss of appetite (26.8% versus 13.2%, P = 0.007). Although not significantly different, seropositive participants also reported more frequently severe, unexpected muscle or joint pain (25.0% versus 15.2%, P = 0.06) and shortness of breath (35.7% versus 24.3%, P = 0.06), but less frequently a sore throat (25.0% versus 34.9%, P = 0.14).

Next, we compared the percentage of seropositive patients in subgroups (Table 3, Table 4). Hospitality staff tested seropositive more frequently compared to hairdressers (14.2% versus 8.0%, respectively), the odds ratio (OR) being 1.9 (95% confidence interval (CI): 1.1–3.4). Seropositive individuals were found in all job functions with > 2 participants. Within other subgroups, notably high seropositivity rates were observed in participants who consumed > 21 alcohol units per week (28.9%), participants with a high education (22.7%) and participants working 8–20 h per week as a hairdresser or hospitality staff (17.6%), while seropositivity rates were low for obese participants (3.1%) and smokers (6.7%; Table 3). In univariable analyses, a high education level (OR 3.0, 95% CI: 1.7–5.6) and increased alcohol use (OR, 7 glasses per week increment: 1.4, 95% CI: 1.1–1.5) were significantly associated with seropositivity, while increased smoking was associated with seronegativity (OR, 5 cigarettes per day increment: 0.6, 95% CI: 0.4–0.9; Table 4).

**Table 3:** Percentage of seropositive participants in the COco-study, by subgroup.

		Number of participants	Seropositive participants	
		**N**	**n**	**%**
All		497	56	11.3
Work Setting	Hospitality personnel	261	37	14.2
	- Owner / manager	109	16	14.7
	- Service	107	16	15.0
	- Receptionist / host	22	2	9.1
	- All-round	6	2	33.3
	- Cook / kitchen staff	14	1	7.1
	- Cleaning	2	0	0.0
	- DJ	1	0	0.0
	Hairdressers	238	19	8.0
	- Owner and/or hairdresser	236	18	7.6
	- Host	1	1	100.0
	- Beautician	1	0	0.0
Sex	Men	137	14	10.2
	Women	360	42	11.7
Age	17–29 years	169	19	11.2
	30–50 years	214	23	10.7
	51–74 years	114	14	12.3
Born in the Netherlands	Yes	471	54	11.5
Household size	1-person	50	6	12.0
	2-persons	141	17	12.1
	3-persons	120	15	12.5
	4-persons	134	11	8.2
	5-persons	38	5	13.2
	>5-persons	14	2	14.3
Education level	Low	88	8	9.1
	Middle	321	28	8.7
	High	88	20	22.7
Financial difficulties	Yes	88	9	10.2
Workplace location	Breda	272	32	11.8
	Roosendaal	83	8	9.6
	Oosterhout	26	4	15.4
	Etten-Leur	25	4	16.0
	Other municipalities	91	6	6.6
Working hours	8–20 h	119	21	17.6
	21–40 h	256	22	8.6
	> 40 h	121	13	10.7
Chronic disease	Yes	143	15	10.5
BMI	Normal weight (BMI < 25.00 kg/m^2^)	288	37	12.8
	Overweight (BMI 25.00–29.99 kg/m^2^)	145	17	11.7
	Obesity (BMI ≥ 30.00 kg/m^2^)	64	2	3.1
Current smoker	Yes	134	9	6.7
	- <10 cigarettes per day	48	5	10.4
	- 10–20 cigarettes per day	78	4	5.1
	- >20 cigarettes per day	8	0	0.0
Current alcohol use	Yes	415	52	12.5
	- 0.5–7 alcohol units per week^a^	222	27	12.2
	- 8–14 alcohol units per week	102	5	4.9
	- 15–21 alcohol units per week	46	7	15.2
	- >21 alcohol units per week	45	13	28.9

Percentages were calculated by dividing the number of seropositive participants by the total number of participants in that specific subgroup (n/N).

a Participants who answered that they drank 1 alcohol unit for <1 day per week, were considered to drink 0.5 alcohol units per week.

**Table 4:** Association between participant characteristics and seropositivity at baseline.

Dependent variable: seropositivity	Univariable	Bivariable	
Variables	OR (95% CI)	Adjusted OR for participants’ characteristics after adjusting for work setting (95%CI)	Adjusted OR for Work setting after adjusting for covariables: Hospitality personnel (ref: hairdressers) (95%CI)
Hospitality personnel (ref: hairdressers)	1.9* (1.1–3.4)	–	–
Female sex (ref: male sex)	1.2 (0.6–2.2)	1.6 (0.8–3.1)	2.2* (1.2–4.1)
Age (10-years increment)	1.0 (0.8–1.2)	1.0 (0.8–1.2)	1.9* (1.1–3.4)
Native Dutch (ref: immigrant)	1.6 (0.4–6.8)	1.6 (0.4–7.1)	1.9* (1.1–3.4)
Household size (1-person increment)	1.0 (0.8–1.2)	1.0 (0.8–1.2)	1.9* (1.1–3.4)
High education level (ref: low/middle)	3.0*** (1.7–5.6)	2.7** (1.4–5.2)	1.3 (0.7–2.5)
Financial difficulties (ref: no financial difficulties)	0.9 (0.4–1.9)	0.8 (0.4–1.7)	2.0* (1.1–3.5)
Workplace location in Breda (ref: outside Breda)	1.1 (0.6–2.0)	1.0 (0.6–1.9)	1.8* (1.0–3.3)
Working hours (8-hours increment)	0.9 (0.7–1.0)	0.9 (0.7–1.0)	2.0* (1.1–3.6)
Chronic disease (ref: no chronic disease)	0.9 (0.5–1.7)	0.9 (0.5–1.7)	1.8* (1.0–3.2)
BMI (5 kg/m^2^ increment)	0.8 (0.5–1.1)	0.8 (0.5–1.1)	1.9* (1.0–3.3)
Current smoker (ref: not currently smoking)	0.5 (0.2–1.0)	0.4* (0.2–0.9)	2.1* (1.1–3.7)
- Smoking quantity (5 cigarettes per day increment)	0.6* (0.4–0.9)	0.6** (0.4–0.9)	2.0* (1.1–3.5)
Current alcohol user (ref: currently no alcohol use)	2.8 (1.0–8.0)	2.6 (0.9–7.5)	1.8* (1.0–3.3)
- Alcohol quantity (7 alcohol units per week increment)	1.3*** (1.1–1.5)	1.3** (1.1–1.5)	1.4 (0.8–2.6)

CI = confidence interval; OR = odds ratio; Ref = reference.

In the second column, odds ratios for the baseline characteristics and their 95% confidence intervals were calculated using a univariable logistic regression model. In the bivariable logistic regression results (columns 3 and 4), column 3 reports the odds ratio for the variable listed in the first column, after adjusting for work setting (hospitality staff/hairdressers). The last column reports the odds ratio for seropositivity for hospitality personnel compared to hairdressers when adjusting for the covariable listed in the first column. E.g., the odds ratio for hospitality personnel to be seropositive was 2.2 (95% confidence interval 1.2–4.1) when adjusting for sex.

The two participants working both as a hairdresser and in the hospitality industry were excluded from these analyses.

*, p < 0.05; **, p < 0.01; ***, p < 0.001.

In bivariable analyses (Table 4), we evaluated the association between participants characteristics and seropositivity, adjusting for work setting (hairdressers versus hospitality staff; middle column), as well as the association between work setting and seropositivity, adjusting for one other covariable per analysis (right column). The association between work setting and seropositivity remained significant in most analyses, except when adjusting for education level (adjusted OR: 1.3, 95% CI: 0.7–2.5) or alcohol quantity (adjusted OR: 1.4, 95% CI: 0.8–2.6). A high education level (adjusted OR: 2.7, 95% CI: 1.4–5.2), smoking (adjusted OR, 5 cigarettes per day increment: 0.6, 95% CI: 0.4–0.9) and alcohol use (adjusted OR, 7 alcohol units per week increment: 1.3, 95% CI: 1.1–1.5) remained significantly associated with seropositivity after adjusting for work setting.

## 4 Discussion

While it is acknowledged that employees in contact professions are at increased risk of being exposed to SARS-CoV-2, most studies evaluating SARS-CoV-2 transmission in contact professions focus on healthcare workers (Sikkema et al., 2020). However, healthcare workers receive training and have experience in using protective equipment, while employees working in non-medical contact-intensive professions do not. Due to this difference, it is important to study non-medical contact-intensive professions separately. As far as we know, the COco-study is the largest cohort study worldwide evaluating SARS-CoV-2 transmission in workers in non-medical contact-intensive professions.

We opted to include hairdressers and hospitality personnel. The hospitality industry is often mentioned as a major source of SARS-CoV-2 transmission (de Gier et al., 2020, Furuse et al., 2020). Considering that hospitality personnel often sees many customers per day in crowded environments, it is conceivable that transmission occurs frequently. Indeed, Fisher et al. concluded that patients who tested positive for SARS-CoV-2 reported to have visited a bar or restaurant more frequently than negative tested patients (Fisher et al., 2020). A Japanese study reported that restaurants and bars were the second most frequent source of clusters (16%), after healthcare facilities (30%) (Furuse et al., 2020). On the other hand, close contact with customers is frequently brief. Furthermore, clusters are easier identified in bars and restaurant as friends and family often meet in groups, facilitating tracing the source of someone’s SARS-CoV-2 infection. Therefore, we wanted to study whether hospitality personnel was actually at increased risk for SARS-CoV-2 infections.

Hairdressers were selected as a second large non-medical, contact-intensive profession. Hairdressers will generally see fewer customers per day than hospitality personnel, but for a longer period of time, being unable to keep physical distancing. Some studies have suggested that hairdressers are at increased risk for COVID-19 (de Gier et al., 2020). However, hairdressers are often behind the customer, reducing the chance that a hairdresser will be in contact with droplets of saliva compared to direct face-to-face contact. Furthermore, another study reported no transmission to customers when two hairdressers with COVID-19 used face masks, but was limited in its size (Hendrix et al., 2020).

We observed that employees in the hospitality industry had significantly more frequently SARS-CoV-2 antibodies than hairdressers (14.2% vs. 8.0%). Additionally, a high education level and increased alcohol use were associated with seropositivity.

During the first wave, the province of North-Brabant had an estimated seroprevalence of 8.4% in June 2020 (Vos et al., 2020). Vos et al. concluded that the seroprevalence in the area of the COco-study was slightly lower than in the rest of the province (Vos et al., 2020). In a second study, seroprevalence was estimated at 6–8% in the study area in May 2020 (Sanquin Research, 2020). We need to be cautious interpreting these findings, as seropositivity rates varied widely between counties in the province, and were amongst others dependent on sex, age, education level and socioeconomic status. However, comparing these percentages to our data, it suggests that the seroprevalence in hairdressers is similar or only slightly higher compared to the general population. Previously, Dutch researchers reported that hairdressers tested more frequently positive for COVID-19 compared to other populations (de Gier et al., 2020). However, that study measured the positive percentages in hairdressers who were getting tested, not the positive percentage in a general population of hairdressers. Additionally, the data of that study was gathered when polymerase chain reaction (PCR) tests were limitless available, while limited in the time period we studied.

The higher seroprevalence rate in hospitality personnel could indicate that they are at increased risk of SARS-CoV-2 at work compared to the general population, similar to other contact professions such as medical staff who do not work with COVID-19 patients (Milani et al., 2021). However, alternative explanations for our study group exist. Two events are thought to have facilitated viral spread in the Netherlands in late February and early March: travelers returning from ski vacations in northern Italy and carnival celebrations. Carnival celebrations in North-Brabant in February resulted in crowded bars, while no measurements were active yet to reduce viral transmission, as these were introduced on March 6 (Streeck et al., 2020). Potentially, the increased seropositivity is a result of this event. Moreover, hospitality personnel might have more social contacts in general, and therefore an increased risk for SARS-CoV-2 outside of work too. The COco-study will enable evaluation of these alternative explanations in the longitudinal analyses. Finally, we observe an association between alcohol use and seropositivity in hospitality staff (OR: 1.3, 95% CI: 1.1–1.5) but not in hairdressers (OR: 1.0, 95% CI: 0.6–1.7; data not shown). The hospitality industry was closed from March 15 to June 1, hairdressers from March 24 to May 11. After reopening, basic preventive measures included physical distancing, not shaking hands and stay at home when experiencing symptoms (NB facial masks were only introduced in the Netherlands in September). Alcohol use might make people less prone to observing physical distancing and other hygienic instructions. However, alcohol use in general reduces inhibitions and facilitates social interaction, which might result in an increased transmission risk.

Smoking was inversely associated with seropositivity, while patients with existing lung conditions have an increased risk for severe COVID-19 (Alqahtani et al., 2020). Various explanations for this discrepancy exist. Smoking generally reduces inflammatory responses and may therefore reduce antibody formation after COVID-19 (Kashyap et al., 2020). Second, smokers may have been warned as additional risks for smokers were communicated early, resulting in more careful behavior. Third, our population was relatively young, smokers may not have developed lung disease yet.

Only a small fraction (19%) of participants who experienced symptoms associated with COVID-19 was seropositive. Since participants only had mild symptoms, it is possible that not everybody who had been infected, developed detectable antibodies (Self et al., 2020, Hou et al., 2020, Lynch et al., 2020). However, symptoms were recorded during a time in which other viruses were circulating, which we consider the primary explanation for the relatively low seropositivity rate. Interestingly, 32% of seropositive participants did not report any symptom in 2020, suggesting asymptomatic infections (Nikolai et al., 2020, Milani et al., 2021). However, this may be an over- or underestimation: we cannot rule out recall bias, but we did not confirm whether participants actually had COVID-19 at the time they experienced symptoms due to the limited availability of PCR tests. Analyses of the longitudinal data of the COco-study will provide more insight into the antibody response duration. Anosmia and/or ageusia were the most important indicators for seropositivity. Previous studies also reported that these two symptoms are characteristic for COVID-19 disease.

The COco-study may assist policymakers making decisions concerning measures to control viral spread. The current study found that hairdressers are not at increased risk for SARS-CoV-2, despite minimal additional hygienic measures. Hence, this industry is likely to contribute minimally to SARS-CoV-2 transmission. Our data suggest that SARS-CoV-2 transmission may be higher in the hospitality industry. Analysis of longitudinal data is needed to evaluate whether measures, such as limited seating and face masks, may eliminate this increased transmission risk in the hospitality industry. We cannot conclude from our current data whether participants were infected during or before/after work. Nevertheless, with these data available, we think that hospitality businesses should be encouraged to take additional measures to prevent SARS-CoV-2 transmission to staff and customers.

This study has several limitations. Due to the sample size, we were limited in conducting multivariable analyses. However, in all bivariable analyses we conducted, the odds ratio remained above 1 for hospitality staff compared to hairdressers, suggesting that hospitality staff had a higher risk for COVID-19 compared to hairdressers regardless of the covariate used. Our study lacks a control group in the general population. Instead, we compared our data to representative measurements in the general population done by other groups. However, creating a control group without confounding will be challenging considering the many factors determining SARS-CoV-2 infection risk. Our study may have been subject to selection bias, but seems to adequately represent an average population of hairdressers and hospitality staff (Supplementary Table 1). For example, 90% of our hairdressers were female, versus 91% of all hairdressers in the Netherlands. The largest discrepancy was in the education level of hospitality workers: more hospitality staff was highly educated in our study population compared to the average population. Most likely this is an error in the way participants answered the questionnaire: many students in our study population considered themselves highly educated while still in college, which would be registered at a lower level in the average population. Finally, due to limited availability of PCR tests in the period before starting the study, we do not have information to objectively confirm whether they had been infected with SARS-CoV-2: only 4 participants had been tested positive by PCR. In the longitudinal analyses, we will be able to compare seroconversion rates to positive PCR test rates, as PCR tests became widely available after the baseline measurement.

In conclusion, we observed that employees in the hospitality industry had significantly more frequently SARS-CoV-2 antibodies than hairdressers. The seroprevalence amongst hairdressers was similar to the seroprevalence as recorded in the general population. Future analyses of longitudinal data in our cohort will allow further evaluation of the role of the hospitality industry and hairdressers in SARS-CoV-2, and which measures successfully restrict transmission in these industries.

## 5 Funding statement

The COco-study receives financial support from the regional public health service (GGD) of West-Brabant and the Dutch National Institute for Public Health and the Environment (RIVM). These are government-funded, non-commercial organizations, and they did not influence any aspect of the content of the study.

## CRediT authorship contribution statement

**Dymphie Mioch:** Conceptualization, Methodology, Investigation, Formal analysis, Writing – original draft. **Sandra Kuiper:** Conceptualization, Methodology, Formal analysis, Writing - review & editing, Funding acquisition. **Wouter den Bijllaardt:** Conceptualization, Methodology, Investigation, Formal analysis, Writing - review & editing. **Cornelia H.M. Jaarsveld:** Conceptualization, Methodology, Formal analysis, Writing - review & editing. **Jan Kluytmans:** Conceptualization, Methodology, Investigation, Formal analysis, Writing - review & editing. **Esther Lodder:** Conceptualization, Methodology, Formal analysis, Writing - review & editing. **Michel D. Wissing:** Conceptualization, Methodology, Investigation, Formal analysis, Writing – original draft, Writing - review & editing, Supervision, Funding acquisition, Visualization.

## Declaration of Competing Interest

The authors declare that they have no known competing financial interests or personal relationships that could have appeared to influence the work reported in this paper.
