# Supplementary material for: SARS-CoV-2 antibodies in employees working in non-medical contact-intensive professions in the Netherlands: Baseline data from the prospective COco-study
Source: Prev Med Rep. 2021 Oct 8;24:101594. doi: 10.1016/j.pmedr.2021.101594 (PMC8498780; doi:10.1016/j.pmedr.2021.101594)
Supplement: Supplementary data 1 [file mmc1.docx]

**Supplementary data COco-study**

**Supplementary methods: Power calculation**

At the time of study design, very limited data was available on seroprevalence in different populations. We estimated that the baseline seroprevalence in June would be 6% in the general population, based on seroprevalence data available from Sanquin in April and May [8, 9]. We estimated a higher seroprevalence in our study population since businesses had been open in late February and early March without any restrictions and during the carnival celebrations, while outbreaks were occurring during this time period. Between March 23th and May 11th, hairdressers had been closed, and between March 15th (6PM) and June 1st, the hospitality industry had been closed. Therefore, we estimated the seroprevalence in our study population to be approximately 8%. Since seroincidence was 6% between March and June, and it was generally expected that the incidence would be lower during the summer, we estimated the seroincidence in September/October (the first follow-up measurement) to be 4%. We considered it clinically relevant if during follow-up the difference between our study population and the general population was 50%, hence if our study population had a seroprevalence of 15% at the first follow-up measurement (or seroincidence of 7%). Using an alpha of 0.05 and power of 80%, the estimated sample size was 398 participants, 199 per group. Jobs in the hospitality industry frequently include temporary jobs, for example students working parttime in a bar or restaurant to cover their expenses, whereas being a hairdresser involves more training and has therefore less turnover. For this reason, the dropout rate was estimated at 10% for hairdressers and 20% for the hospitality industry. Adjusting for these dropout rates and the baseline seroprevalence rate, the total number of participants required was 498, of whom 238 hairdressers and 260 hospitality staff. This power calculation was based on one follow-up measurement, since continuation of the study would depend on these first results: only if the first follow-up measurement indicated higher seroincidence rates in hairdressers and/or hospitality staff compared to the general population, additional follow-up measurements would be conducted.

**Supplementary methods: Questionnaire**

1. **In what industry do you work?**
   - Hairdresser
   - Hospitality industry
   - Hairdresser and hospitality industry
2. **Per industry, do you have a contract with a fixed number of hours or with flexible hours per week?**
   - Fixed hours
   - Flexible hours
3. **Per industry, how many hours do you work per week (on average)?** I work (on average) … hours per week
4. **What is your job position within the hospitality industry?**

- Service
- Cook
- Working in the kitchen
- Receptionist
- Host/hostess
- Administrative assistant
- Cleaning
- Manager/owner of a restaurant/café
- Manager/ owner of a hotel
- Otherwise, namely…

1. **Wat is your job position within the hairdresser industry?**

- Hairdresser
- Manager/ owner of a barbershop
- Administrative assistant
- Host/hostess
- Otherwise, namely…

1. **What is the four digit postal code of your work location?**

……

1. **What is your year of birth?**

……

1. **What is your gender?**

- Man
- Woman
- Otherwise, namely…

1. **What is your household situation?** *You may choose multiple answers.*

- I live with a partner/spouse
- I live with children under 18 years
- I live with children aged 18 years or older
- I live with my parents
- I live with another adult/other adults
- I do not live with a partner, but I do have a permanent relationship
- I live by myself

1. **How many people live in your household including yourself?**

**......**

1. **What is your highest completed level of education?**

- No education
- Primary education
- Secondary education: Pre-vocational education and training (level 1)
- Secondary education: Pre-vocational education and training (level 2, 3, 4) & Vocational education and training (VET; level 1)
- Secondary education: Vocational education and training (VET; level, 2, 3, 4)
- Secondary education: senior general secondary education, university preparatory education
- Higher professional education (HBO)
- College degree or more (bachelor’s or master’s degree, Ph.D. etc.)

1. **In the past 12 months, did you have financial difficulties?**

- No
- No, but I have to be careful with my expenses
- Yes, some financial difficulties
- Yes, great financial difficulties

1. **What is the country of birth for yourself, your mother and your father?**

| **Yourself** |  |
| --- | --- |
| **Your mother** |  |
| **Your father** |  |

1. **Have you had any of the following symptoms since the start of 2020?** *You may choose multiple answers.*

| Objectively recorded fever | **□** |
| --- | --- |
| Feeling feverish | **□** |
| Coughing | **□** |
| Runny nose / nasal congestion | **□** |
| Shortness of breath | **□** |
| Pain during breathing | **□** |
| General malaise | **□** |
| Fatigue | **□** |
| Severe, unexpected muscle / joint pain | **□** |
| Headache | **□** |
| Sore throat | **□** |
| Diarrhoea | **□** |
| Gastrointestinal symptoms | **□** |
| Loss of appetite | **□** |
| Confusion / irritability | **□** |
| Anosmia/ageusia | **□** |
| Otherwise, namely | **□** |

1. **Have you been tested for COVID-19?**

- Yes
- No

1. **When were you tested for COVID-19? [date]**
2. **Did you have COVID-19?**

- Yes, this is confirmed by a test
- I have been tested because of symptoms, but the test result was negative (no COVID-19)
- I have been tested while not having symptoms. The test result was negative (no COVID-19)
- I have had symptoms that were potentially related to COVID-19, but I have not been tested
- I did not have any symptoms and was not tested.

1. **Have you been hospitalized because of COVID-19 or COVID-19 related symptoms?**

- No
- Yes, duration: … days

1. **Do you have any chronic disease?** *You may choose multiple answers.*

- Lung disease, such as COPD / asthma
- Cardiovascular disease, such as high blood pressure
- Type 1 diabetes
- Type 2 diabetes
- An immune disorder, such as HIV, leukemia, chronic treatment with corticosteroids, splenectomy
- Auto-immune disease, such as rheumatism, MS, Chron's disease
- Cancer, namely:…
- I have another chronic disease, namely…
- None of the above

1. **Which medicines do you use? Include the dosage. [Open question]**
2. **What is your length (without shoes)? [centimeters]**
3. **What is your weight (without clothes)? [kilograms]**
4. **Do you smoke? Smoking includes all types of tobacco products (cigarettes, cigars, etc.), but not the use of an electronic cigarette.**

- No
- Yes, on a daily basis
- Yes, I smoke occasionally
- I used to smoke, but I have stopped since: …

1. **How may cigarettes do you smoke on average per day?**

- Number of cigarettes per day…
- Number of shag per day…
- Number of cigars per day…
- Number of pipes per day…

1. **On a weekday (Monday to Thursday), how frequently do you drink alcohol on average?**

- 4 days
- 3 days
- 2 days
- 1 day
- Less than 1 day
- I never drink on a weekday

1. **How many glasses do you drink on average on a weekday (Monday to Thursday)?**

- 16 or more glasses
- 11-15 glasses
- 7-10 glasses
- 6 glasses
- 5 glasses
- 4 glasses
- 3 glasses
- 2 glasses
- 1 glass

1. **On a weekend day (Friday to Sunday), how frequently do you drink alcohol on average?**

- 3 days
- 2 days
- 1 day
- Less than 1 day
- I never drink alcohol in the weekend

1. **How many glasses do you drink on average on a weekend day (Friday to Sunday)?**

- 16 or more glasses
- 11-15 glasses
- 7-10 glasses
- 6 glasses
- 5 glasses
- 4 glasses
- 3 glasses
- 2 glasses
- 1 glass

*Take in mind a normal day in the past months. Can you indicate how many days per week you performed the activities below and how much time you spent on average on such activity per day? If you have not performed an activity, enter 0.*

| 1. **Commuting (return trip)** |  | | |  | |
| --- | --- | --- | --- | --- | --- |
|  | **Number of days per week** | | | **Average time per day** | |
| Walking from/to work or school | days | | | hour   minutes | |
| Cycling from/to work or school | days | | | hour   minutes | |
|  |  | | |  | |
| 1. **Physical activity at work or school.** | | | | | |
|  |  | | | **Number of hours per week** | |
| Light and moderately strenuous work (for example office work with occasional walking, or work that involves movement with lightweight objects) | | | | hours | |
| Strenuous work (walking or regularly lifting heavy objects while working) | | | | hours | |
|  | | | |  | |
| 1. **Household activities.** | | | | | |
| **Number of days per week** | | | | | **Average time per day** |
| Light and moderately strenuous work (working in a standing position, such as cooking, doing the dishes, ironing, feeding / bathing the child, and activities such as vacuuming/shopping) | | | days | | hours   minutes |
| Strenuous work (such as scrubbing floors, beating carpets, walking with heavy groceries) | | | days | | hours   minutes |
| 1. **Leisure** | | **Number of days per week** | | | **Average time per day** |
| Walking | | days | | | hour   minutes |
| Cycling | | days | | | hour   minutes |
| Gardening | | days | | | hour   minutes |
| Do-It-Yourself (DIY) | | days | | | hour   minutes |
|  | |  | | |  |
| 1. **Sport** (Write down a maximum of 4 sports; for example: fitness training, tennis, running, soccer).   *If you don't play sports, you can skip the question.* | | | | | |
|  | | **Number of days per week** | | | **Average time per day** |
|  | | days | | | hour  minutes |
|  | | days | | | hour  minutes |
|  | | days | | | hour  minutes |
|  | | days | | | hour  minutes |

1. **Are you outside for at least 15 minutes per day between 11:00 and 15:00, with at least your head and hands uncovered?**

- Yes
- No

1. **Have you used a dietary supplement that contains vitamin D in the past three months?**

- Yes
- No

1. **How often do you use a dietary supplement that contains vitamin D?**

- On a daily basis
- Otherwise, namely… a week

**Supplementary discussion:**

**Supplementary table 1.** Dutch population of hairdressers and hospitality staff, as recorded by Statline (a Dutch governmental institution).

|  |  | Dutch hospitality industry  (N=337,000) | | Dutch hairdresser industry  (N=80,000) | | |
| --- | --- | --- | --- | --- | --- | --- |
|  |  | n (x1,000) | % | n (x1,000) | % | |
| Sex | Men | 160 | 47.5 | 7 | | 8.8 |
|  | Women | 176 | 52.2 | 73 | 91.3 | |
| Age (in years) | - 15 - 25 years | 167 | 49.6 | 11 | 13.8 | |
|  | - 25 - 45 years | 86 | 25.5 | 35 | 43.8 | |
|  | - 45 - 75 years | 83 | 24.6 | 35 | 43.8 | |
| Education level | - Low | 115 | 34.1 | 15 | 18.8 | |
|  | - Middle | 177 | 52.5 | 56 | 70.0 | |
|  | - High | 37 | 11.0 | 8 | 10.0 | |

Source: <https://opendata.cbs.nl/statline/#/CBS/nl/dataset/82808NED/table?ts=1626702890665>
